# Self-management strategies and care needs of patients with persistent depressive disorder and their informal caregivers: a multi-perspectives qualitative interview study

**DOI:** 10.3389/fpsyt.2025.1505396

**Published:** 2025-06-20

**Authors:** Ericka C. Solis, Ingrid V. E. Carlier, Noëlle G. A. Kamminga, Albert M. van Hemert

**Affiliations:** ^1^ Department of Psychiatry, Leiden University Medical Center, Leiden, Netherlands; ^2^ Department of Psychiatry and Medical Psychology, Maastricht University Medical Center, Maastricht, Netherlands; ^3^ Faculty of Health, Medicine and Life Sciences (MHeNs), Maastricht University, Maastricht, Netherlands

**Keywords:** self-management, chronic depression, persistent depressive disorder, qualitative study, care needs, carer, informal caregiver

## Abstract

**Introduction:**

When patients with persistent depressive disorder (PDD) respond insufficiently to available evidence-based treatments, depression treatment guidelines recommend psychiatric rehabilitation through self-management. Preferably, the intervention should involve the patient’s informal caregiver.

**Methods:**

To gain insight into the healthcare needs of PDD patients and their caregivers and to facilitate the implementation of a self-management program, we conducted individual semi-structured interviews with 28 PDD patients and 9 informal caregivers regarding their self-management/coping and needs. Transcripts were analyzed with Grounded Theory using three sensitizing concepts (PDD experience, self-management/coping, needs).

**Results:**

Patients had 9 main themes and caregivers had 11 main themes. Patients and caregivers shared 9 main themes, pertaining to powerlessness, patients’ identity changes, shame/stigma, relationship dissatisfaction, family suffering, self-management attitudes, self-management strategies, coping support, and coping complications. While self-management attitudes of patients were mixed, those of caregivers were positive. Care needs of both groups centered on psychoeducation and communication skills development. Caregivers reported urgently needing support in dealing with patients’ suicidal behavior.

**Discussion:**

Our findings underscore the profound burden of PDD on both patients and their informal caregivers. We strongly recommend that healthcare professionals encourage and facilitate the development of self-management in depressed patients early in the treatment process and involve informal caregivers, particularly within suicide prevention strategies.

**Clinical Trial Registration:**

https://onderzoekmetmensen.nl/en/trial/55681, Netherlands Trial Register Identifier NL5818.

## Introduction

Depression is a highly prevalent and disabling chronic disease (1, 2), associated with increased mortality, suicide risk, and economic burden (3–5), in addition to a substantially decreased quality of life for both the individual and their relatives (6). Even after diagnostic remission, subclinical symptoms often persist, and relapse is common (7, 8). The relapse risk increases with each subsequent depression episode, leading to long-term recurrent depression (9). Additionally, 10 to 17% of patients continue to meet depression criteria for more than 2 years (2). Depression with such a long-term duration (>2 years) is referred to as persistent depressive disorder (PDD) in the latest Diagnostic and Statistical Manual, 5^th^ revision (DSM-5-TR) (10). It can also be referred to as chronic depression. PDD has a lifetime prevalence of 1 to 6% (11, 12).

Between 30-50% of patients with PDD respond insufficiently to antidepressant or psychological treatment (13–15), despite intensive or prolonged care (16). Thus, instead of supportive non-protocolized care, the revised multidisciplinary guidelines for depression treatment (17) recommend psychiatric rehabilitation as the next step for PDD in patients with inadequate therapeutic results. There is growing consensus that treatment in specialized mental healthcare should move its focus from symptomatic recovery to functional recovery for chronic disorders including PDD (15). Rather than reducing depressive symptoms, functional recovery focuses on the restoration or enhancement of the patient’s ability to function effectively in various life domains. Patients with chronic disorders are encouraged to believe that, despite their limitations, they can live fulfilling lives and contribute meaningfully to society (18). Patients, thus, learn to cope in a healthier way with their PDD/chronic depression. This concept of coping is crucial to psychiatric rehabilitation. It encompasses a wide range of cognitive and behavioral efforts to manage the (consequences of a) disease, such as actively seeking support and mindfully changing thought patterns. Related to coping, moreover, is the concept of self-management. Self-management is a subset of coping that refers to the organized and proactive effort of the individual to monitor, assess, and manage cognitions, emotions, and behaviors related to living with a (chronic) disease, with the goal of establishing a dynamic and continuous process of self-regulation (19).

Rehabilitation through self-management has been previously established for chronic somatic diseases and for severe psychiatric illness, such as schizophrenia/psychosis (20, 21). However, it is still developing for chronic depression and chronic anxiety. For chronic depression, self-management focuses on symptom stabilization, enhancing autonomy, improving psychosocial functioning, boosting work/life functioning (e.g., participating in civic activities, going to school/(volunteer)work) and the prevention of relapse and suicide) (22). While some studies show that optimal self-management improves quality of life and health outcomes (19), results have been mixed (23, 24). Also, it is recommended to involve the patient’s life partner or informal caregiver (further referred to as “caregiver”) in treatment. This may enhance patient outcomes and improve self-management (25–28), although this is still not common practice in specialized mental healthcare.

Moreover, caregivers may also require support in mental healthcare considering the crucial role they play in caring for patients in terms of medication and treatment compliance (29). Caring for someone with (chronic) depression has been compared to living with someone with other severe psychiatric illness, such as schizophrenia (29–31). Similar to the situation of those caring for persons with schizophrenia, caring for someone with PDD can present a high level of burden. This includes emotional, psychological, social and financial problems for the caregiver (32). Involvement of the caregiver (of patients with severe psychiatric illness) in psychoeducational interventions and cognitive behavioral family interventions has been shown to improve the patient’s psychiatric symptoms as well as the caregiver’s perception of caregiving, perceived burden, and expressed negative emotion (33, 34). Involving the caregiver in interventions may, thus, strengthen the caregiver and the patient/family system (35, 36).

Greater insight into the experience, utilization, and optimization of self-management for patients with PDD, through qualitative research, may contribute to effective implementation of self-management in mental health clinics. Considering the complex, non-linear nature of recovery (37), conducting qualitative/mixed-methods research would allow us to contextualize and further understand quantitative research findings for patients and their caregivers. It is also important to understand how caregivers acquire knowledge and implement skills for their own health management and that of the patient for whom they provide care (38).

Previous qualitative studies have primarily examined self-management in adults with acute depressive disorder (39–45), in (older) persons with (subclinical) depression (46–50), and in adults with chronic somatic disorders with comorbid depression (51–54). Only a handful of studies have focused on self-management in patients with PDD/chronic depression in secondary mental healthcare (55–57). These latter studies stressed the importance of the patient’s empowerment and acceptance of the disorder. They also call for further research to illuminate hindering factors in coping with chronic depression, as viewed from multiple perspectives (i.e. patients, informal caregivers, healthcare providers) (55, 56).

To date, no qualitative study has been conducted pertaining to self-management and care needs for PDD patients and their informal caregivers together. Further qualitative data is required to guide implementation of- and/or improve self-management programs for patients with PDD that include informal caregivers. Therefore, this qualitative study aimed to understand how patients with PDD and their caregivers experience/cope with PDD and identify their healthcare needs and potential challenges to meeting them.

## Materials and methods

### Study design

This qualitative interview study was nested in a mixed-methods, pragmatic randomized controlled trial (RCT) that took place in several Dutch specialized outpatient clinics (see Acknowledgements) between April 2017 and March 2022. In the RCT, a self-management program (“*Patient and Partner Education Program for All Chronic Diseases-adapted for Persistent Depressive Disorder”*, PPEP4All-PDD) was evaluated against care as usual (CAU, without caregiver involvement) in patients with PDD. If patients were allocated to PPEP4All-PDD, caregivers were invited to participate in the project. PPEP4All-PDD, consisting of nine sessions that were offered in separate patient and caregiver groups or individual sessions.

Inclusion criteria for patients were a PDD diagnosis (based on clinician referral and confirmed using the Mini-International Neuropsychiatric Interview (MINI) (58, 59); age of 18 years or older; and treatment indication for psychiatric rehabilitation (confirmed by the treating clinician, and defined by multidisciplinary depression-guidelines (60) as having had at least one (unsuccessful) previous psychological treatment and at least two (unsuccessful) previous medication trials). Exclusion criteria for patients were severe psychopathology (e.g., schizophrenia); acute suicide risk; severe disabling somatic disorders; severe cognitive problems; current active psychotherapy; and insufficient Dutch fluency. Inclusion criteria for caregivers include the ability to participate in PPEP4All-PDD (minimum 3 sessions, for pragmatic reasons) and not currently receiving active psychotherapy. All participants provided written informed consent for each part of the study.

The study was approved by the Medical Ethical Committee (MEC) of the Dutch Leiden University Medical Center (LUMC). The main research center was the Department of Psychiatry of the LUMC. The detailed methodology, design, and results of the RCT study have been reported elsewhere (24, 61).

### Participants and recruitment for the qualitative interview study

Patients and caregivers who gave consent to be approached for the nested qualitative study were invited by telephone to participate in an individual semi-structured qualitative interview. For patients, we aimed for a purposive sample, with maximum variation on age, gender, education level, marital status, and baseline depression severity (62, 63). For informal caregivers, we aimed to include variation on age (adults > 18), gender, education, and relationship to the patient (e.g., life partner/spouse, daughter, brother). Most qualitative interviews took place prior to the start of the self-management intervention with the exception of a few participants who could not be reached earlier. Participants received a €20 gift card for the interview.

### Data collection of the qualitative study

Individual in-depth semi-structured interviews took place at participant’s home, LUMC or by telephone. During the COVID-19 pandemic, the interviews took place exclusively by telephone. A team of six psychology research assistants and the first author (ES) conducted the interviews in Dutch with the participants.

For each interview, we used a topic guide, which was initially evaluated in an unpublished pilot study of patients with PDD. The topic guides for patients and caregivers were partly based on Chambers et al. (55) and used sensitizing concepts as starting points to draw attention to important aspects/topics of both the RCT and the qualitative study (64). The topic guide for patients (see **Table A of the Supplementary Material**) concerned three sensitizing concepts: (A) lived experience with depression, (B) coping with chronic depression/self-management, and (C) needs for care. The topic guide for caregivers (see **Table B of the Supplementary Material**) concerned four sensitizing concepts: (A) lived experience dealing with a loved one with depression, (B) helping a loved one with depression, (C) coping/self-management with chronic depression of the patient, and (D) needs for care. The topics/questions were presented to the participant as they naturally came up during the interview, to maintain the flow of the interview.

At the beginning of the interview, participants gave verbal consent for audio recording and transcription of the interview. The interviews were recorded and then transcribed verbatim by research assistants using the online Transcribe application (https://transcribe.wreally.com). Patient transcripts were verified for errors and completion with the audio recording by a research assistant. Personal identifiers were removed, and transcripts were saved under a participant code. If information was unclear or if requested by the participant, the transcript was sent to the participant for verification and then amended according to the participant’s comments (i.e., respondent validation). The patient interviews were between 45 and 90 min, and partner interviews were between 30 to 80 min. An audit trail of each interview was created. We took notes during and after the interviews regarding our impressions or interpretations that may help us interpret the data. Memo writing was utilized throughout the analytic process to track possible interpretations and final decisions. Data collection was complete when data saturation was reached.

### Theoretical framework of the qualitative study

Data were analyzed using Grounded Theory (GT), which aims to construct new theories or rationales grounded in data through an inductive, bottom-up approach (65–68). In accordance with GT, we used the constant comparative method, where data collection and analysis occur in tandem. In general, our interviews were dynamic in structure (i.e., semi-structured, maintaining interview flow) and in content (i.e., asking additional questions, probing questions) (69). We created notes and memos of interviews, and additional questions were added to the interviews after the first round. Data interpretation and analysis were then completed after all data were collected (i.e., after the second round).

### Data coding and analysis of the qualitative study

We used Atlas.ti version 23 software (Atlas.ti Scientific Software Development GmbH) to code the qualitative data. In accordance with GT, we used open coding, axial coding and selective coding (see Supplementary Material about coding) while considering our sensitizing concepts. Through this stepped analysis process of interpreting the data, thematically-connected (sub)groups of codes emerged from interview transcripts, resulting in sub-, main, and core- themes for patients and caregivers. Interviews were independently coded by a research assistant and the first author (ES). Codes were discussed at the two rounds of interviews, to conduct member checks. Throughout the process, we focused on thinking theoretically. Despite the team’s background in psychology, the researchers remained open an flexible to the data, being both creative and sensitive and allowing the data to lead to any unexpected insights (70, 71). The interview transcripts were then analyzed by the first author (ES); emerging themes remained grounded in the data. Finally, through a process of constant comparison, results between patients and caregivers were compared and arranged into a model using the sensitizing concepts. The second author (IC) functioned as auditor throughout the coding/analysis process (72).

In line with multi-perspective interviewing, the coding for patients and caregivers were performed separately (73). During the analysis process, the results of patients and caregivers were triangulated by comparing, contrasting, and integrating the themes (73). We focused on similarities and differences of patients and caregivers as groups, rather than on patient-caregiver dyads. In our results, we aimed to maintain separate patient and caregiver themes and to present similarities and differences among the patient and caregiver themes.

### Validity of the qualitative study

Care was taken to ensure the participant’s experiences and perspectives were accurately received and appropriately interpreted, taking into account potential researcher bias (74, 75). Rigorous methodological checks, such as member checking, peer debriefing, third-member auditing (through referential adequacy), and audit trial/memo utilization, were used to increase trustworthiness and credibility, within the framework of GT (74). In addition, we selected our participants with the aim of increasing the chance of shedding light onto our research question (i.e., credibility) (75). Thus, we included patients with PDD-diagnosis and continued symptoms despite previous treatment, with consideration to variation in patient characteristics. We also included informal caregivers to consider another perspective on the situation. Moreover, we ensured that the codes we selected as meaning units were sufficient in terms of length to ensure comprehension and relation to the data (75). In terms of transferability of results to other settings or groups (74, 75), we have provided contextual descriptions of findings. This includes the culture and context (i.e., outpatient specialized mental healthcare in the Netherlands, at a time when self-management was being implemented, see also **Table 1** for patient characteristics); selection and characteristics of patients; data collection; and analytical process. For the latter, please see ‘Data collection’ and ‘Data coding’).

**Table 1:** Sociodemographic and clinical characteristics of participants.

Sociodemographic characteristics	Patients (n = 28)	Caregivers (n = 9)
Age		
Mean (SD)	50.0 (10.2)	60.6 (15.1)
Range	40-77	26-74
Gender		
Female, n (%)	18 (64.3%)	5 (55.6%)
Male, n (%)	10 (35.7%)	4 (44.4%)
Ethnic background^a^		
Dutch, n (%)	26 (92.9%)	7 (77.8%)
Other, n (%)	2 (7.1%)	1 (11.1%)
Education level^b^		
Lower education, n (%)	6 (21.4%)	3 (33.3%)
Higher education, n (%)	22 (78.6%)	5 (55.6%)
Employment status		
Employed, n (%)	1 (3.6%)	2 (22.2%)
Unemployed/retired, n (%)	27 (96.40%)	6 (66.7%)
Marital status		
Married/cohabitating, n (%)	11 (39.3%)	8 (88.9%)
Other (single, widowed, divorced), n (%)	17 (60.7%)	1 (11.1%)
Caregiver relationship		
Spouse/life partner, n (%)	–	6 (66.7%)
Other (e.g., mother, daughter)*, n (%)	–	3 (33.3%)

Clinical characteristics	Patients (n = 28)	Caregivers (n = 9)
Clinical psychopathology present, SQ-48-Total, n (%)^c^	22 (78.6%)	–
Comorbid clinical anxiety present, SQ-48-Anx, n (%)^d^	17 (60.7%)	–
Diagnosis, PDD, MINI, n (%)^e^	28 (100%)	–
Age of onset of depression, MINI, mean (SD)^f^	32.9 (18.1)	–
Number of lifetime depressive episodes, MINI mean (SD)^g^	9.0 (12.1)	–
Severity of depression, MINI, n (%)^h^		
Mild, n (%)	2 (7.1%)	–
Moderate, n (%)	16 (57.0%)	–
Severe without psychotic features, n (%)	6 (21.4%)	–
Severe with psychotic features, n (%)	2 (7.1)	–
Suicide risk, MINI^h^		
Low, n (%)	21 (75.0%)	–
Moderate, n (%)	3 (10.7%)	–
High, n (%)	4 (14.3%)	–

“Caregivers” refers to romantic partners or other informal caregivers. Clinical characteristics were not available for caregivers. There was 1 missing value for caregivers for the following sociodemographic characteristics: ethnic background, education level, and employment status.

A*bbreviations*. SQ-48 = Symptom Questionnaire 48; SQ-48-Anx = Symptom Questionnaire 48-Anxiety subscale; PDD = Persistent Depressive Disorder; MINI = Mini-International Neuropsychiatric Interview.

* Other caregiver relationships included 1 mother, 1 daughter, and 1 friend.

a Dutch ethnic background was assumed when the patient and both parents were born in the Netherlands.

b Lower education was defined as having completed elementary school, lower general primary education or no education at all, whereas higher education was defined as having more than lower education and included university studies.

c Based on the SQ-48 total score. Clinically-relevant cut-off score is 42 (Carlier et al., 2012A; Carlier et al., 2012B; Carlier et al., 2017).

d Based on the SQ-48-Anx subscale score. Clinical-relevant cut-off score is 11 (Carlier et al., 2012A; Carlier et al., 2012B; Carlier et al., 2017).

e Patient referred to by treating clinician. Diagnosis confirmed using the MINI. Diagnosis is also referred to “chronic depression” in this study.

f Age of onset as reported on the MINI.

g Number of depressive episodes as reported on the MINI, *n* = 25: 1 patient with extreme response removed (=99), and 2 patients did not complete this question.

h Suicide risk based on the MINI.

## Results

### Sociodemographic and clinical characteristics

The qualitative interview study included 28 patients (PPEP4All-PDD, *n* = 16; CAU, *n* = 12) and 9 caregivers (PPEP4All-PDD, *n* = 8; CAU, *n* = 1). Participant characteristics are described in **Table 1**.

There were 18 female and 10 male patients, ranging in age from 40 to 77, with an average of 50 years old. Most were Dutch (92.9%), unemployed/retired (96.4%), and had completed higher education (78.6%). Based on the MINI, patients had a PDD/chronic depression diagnosis and an average age onset of depression at 33 years old, with an average of 9 previous lifetime depressive episodes. There were 4 patients who reported a high, but not acute suicide risk on the MINI and 8 with a severe level of depression (with or without psychotic features). Looking at the Symptom Questionnaire-48 (SQ-48) (76–78), 78.6% of patients experienced comorbid clinical psychopathology (SQ-48 total) and 60.7% of patients had comorbid clinical anxiety (SQ-48 subscale). Among the caregivers, there were 5 women and 4 men, ranging in age from 26 to 74 years old with an average of 61 years old. Most were Dutch (77.8%), unemployed/retired (66.7%), life partners/spouses (66.7%), and had completed higher education (55.6%). Considering other patient and caregiver relationships (33.3%), there was a mother (*n* = 1), daughter (*n* = 1), and close friend (*n* = 1) in the study. Clinical variables were not available for caregivers.

### Overview of the emerging themes in patients and caregivers

**Figure 1:**
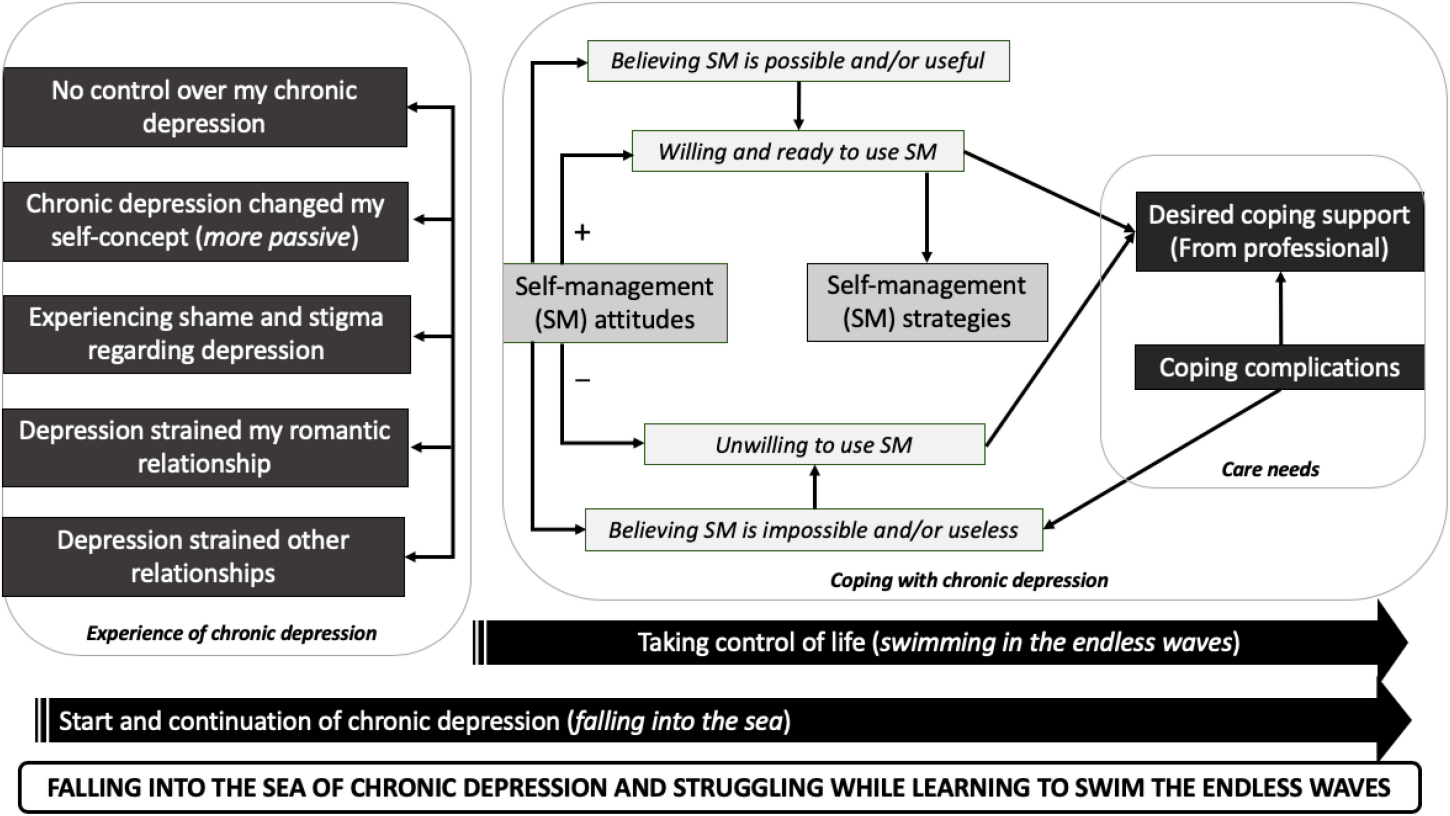
Patient themes. SM, Self-management.

For patients and caregivers, emerging (core-/main-/sub-)themes were arranged according to our pre-existing sensitizing concepts. For patients, we identified 44 subthemes that were aggregated into 9 main themes (see **Table C in the Supplementary Material**, which shows all themes, including examples of codes and quotations). We summarized the patient core theme as “*Falling into the sea of chronic depression and struggling while learning to swim the endless waves.*” This core theme uses a poignant metaphor, portraying chronic depression as a “sea with endless waves.” It encompasses the negative changes patients undergo in the development of (chronic) depression and the process of learning to cope with it. **Figure 1** illustrates how patient main themes interconnect to support the patient core theme.

**Figure 2:**
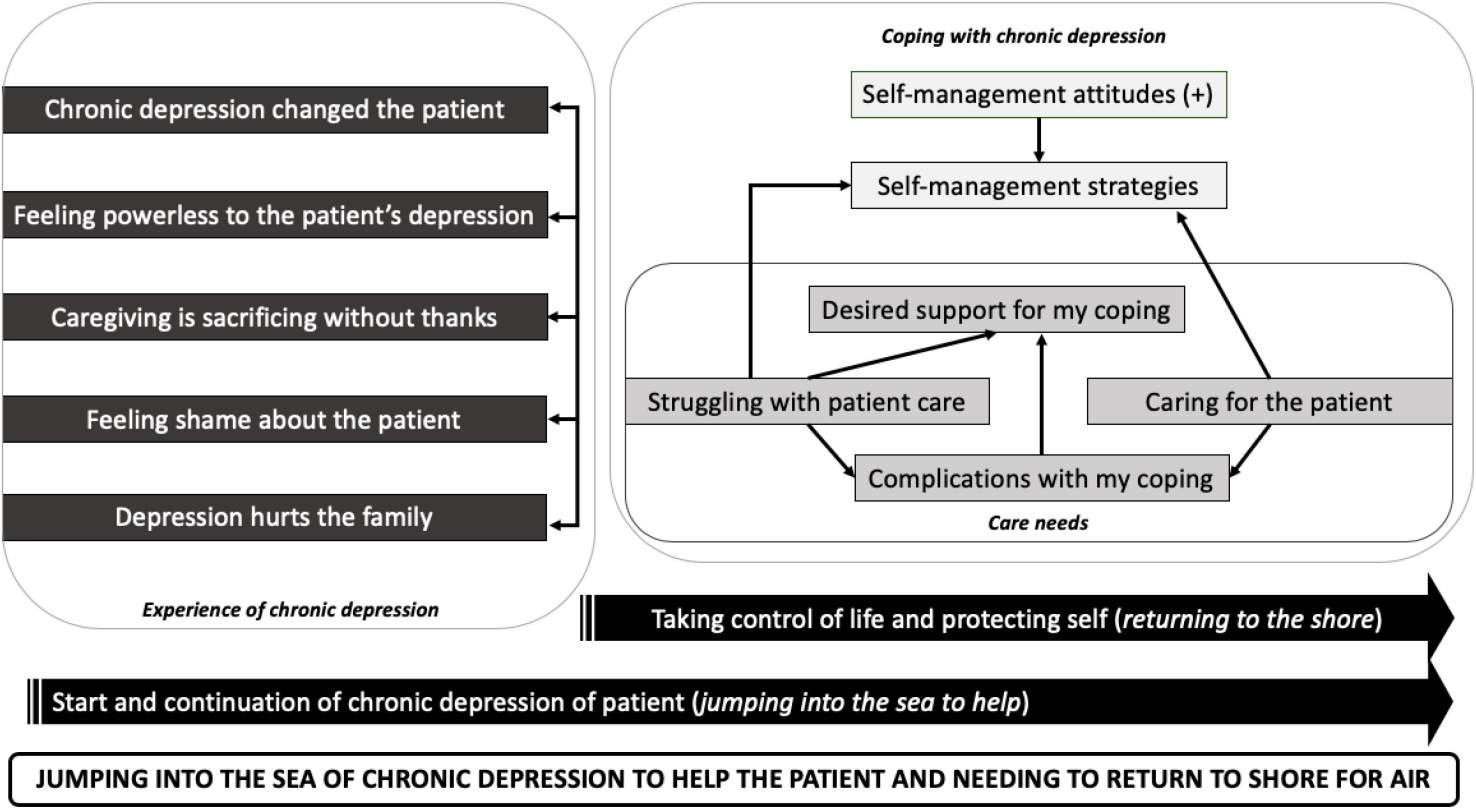
Caregiver themes.

For caregivers, we identified 32 subthemes that were aggregated into 11 main themes (see **Table D in Supplementary Material**, which shows all themes, including examples of codes and quotations). We summarized the caregiver core theme as “*Jumping into the sea of chronic depression to help the patient and needing to regularly return to shore for air.”* This core theme reflects the long-term efforts exerted by the caregiver to help the patient with chronic depression and dealing with the fear of the patient drowning in the water of chronic depression. This long-term exertion, similar to when one swims for a long duration, demands taking distance from the situation and recharging one’s energy (i.e., going to shore for air). Additionally, this metaphor reflects the feeling of being forced to help the patient: the caregiver may have difficulty understanding why the patient cannot escape the sea, and there may be a fear that the patient may drown if the caregiver does not continue to assist. While the caregiver could choose to leave, there may be internal struggle, and this choice becomes more complicated when children are involved. **Figure 2** illustrates how caregiver main themes interconnect to support the caregiver core theme.

### Comparison of main themes between patients and caregivers

**Figure 3:**
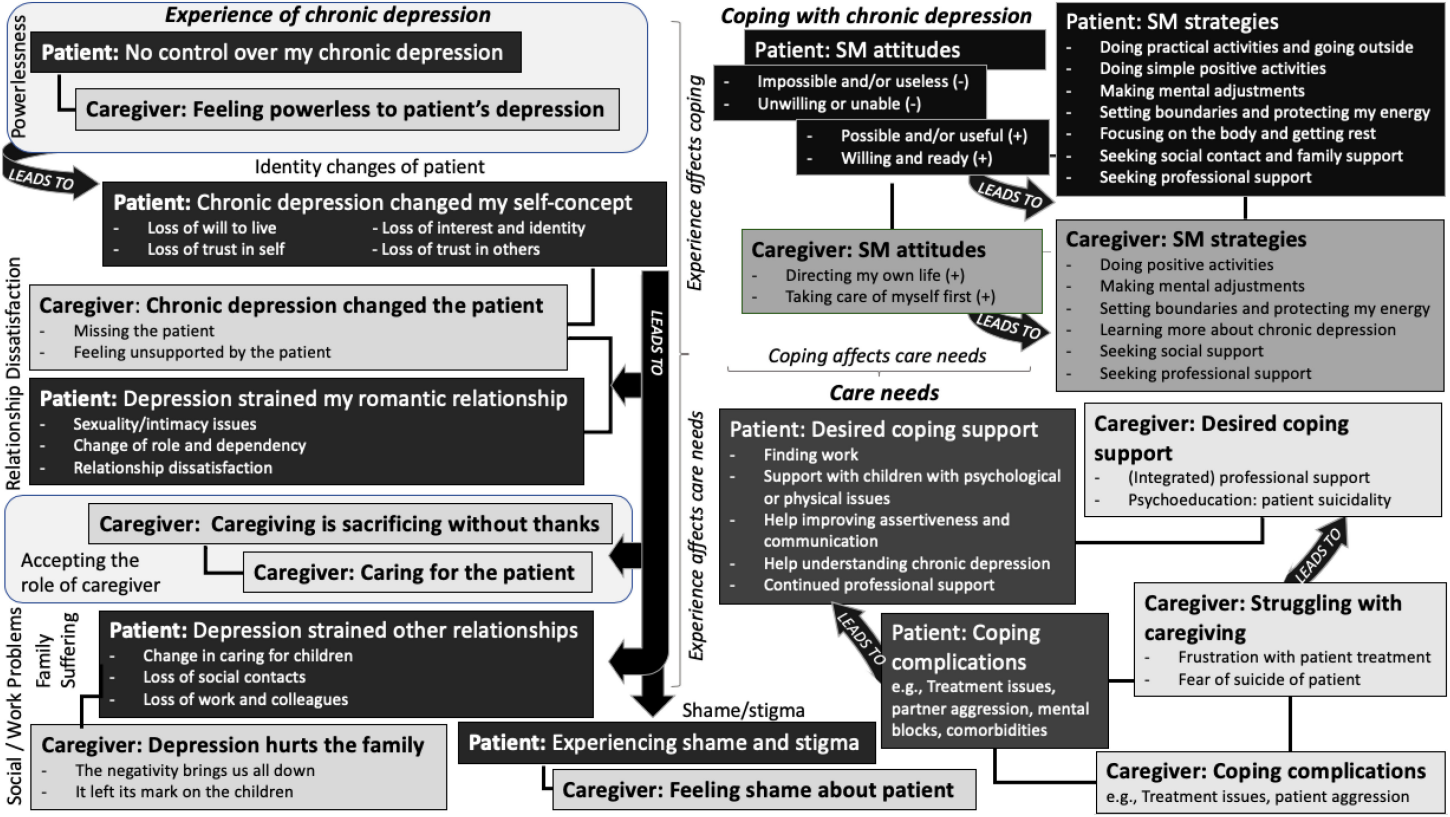
Comparison of main themes showing links between similar themes of patients and those of partners/caregivers.

*Similarities:* Eight patient main themes and eight caregiver main themes (i.e., the same issues but formulated from the caregiver perspective) showed significant thematic overlap. These shared main themes pertained to powerlessness, self-concept/identity changes (of the patient), shame/stigma, relationship dissatisfaction, family suffering, self-management attitudes, self-management strategies, coping support, and coping complications. **Figure 3** shows which main themes of patients and caregivers are similar/related (indicated by connecting line).

*Differences:* Although the concept of “family suffering” was shared by patients and caregivers, one theme related to issues unique to the patient regarding social and work problems (“Depression strained other relationships”). For caregivers, there were 3 unique themes regarding accepting the role as caregiver (“Caregiving is sacrificing without thanks”, “Caring for the patient”, “Struggling with caregiving”). Key differences between patients versus caregivers were as follows: (a) patients struggled to identify a hopeful vision of the future; (b) patients expressed lowered capacity for childcare; (c) patients expressed needing more help with dealing with children with psychological problems; (d) patients expressed having more problems with their social network; (e) patients experienced more work-related problems; (f) patients struggled to understand and translate concepts of recovery and self-management in their lives; (g) caregivers had more positive attitudes about self-management; (h) the patient’s suicide risk was the most important concern for caregivers; and (i) caregivers expressed needing broader psychoeducation than patients, which included information on both chronic depression and suicidality. The main themes of patients and caregivers, in relation to the sensitizing concepts, are compared and explained in detail below (see **Figure 3** for the comparison of main themes):

### Experience of chronic depression

Powerlessness: Patients and caregivers described depression as sudden and overwhelming, leading to feelings of hopelessness and powerlessness. For patients, this powerlessness was associated with lack of insight (i.e., self-knowledge regarding personal depressive cues), while for caregivers, it was related to a lack of knowledge about depression (e.g., course of depression and suicidal behavior). The perceived risk of suicide and unpredictable nature of depression heightened caregivers’ distress, often necessitating pharmacological and psychological treatment to manage their own emotional symptoms.

Patient: *“I can’t recognize when I start getting depressed again. It’s very difficult, but you can’t do anything about it.” (P.4, female, single, 72 years old)*

Caregiver: *“It’s difficult, and what makes it difficult is that it is really unpredictable.’” (C.8, female, life partner/spouse, 74 years old)*

Identity Changes (of the patient): Patients experienced a loss of motivation and hope for the future, leading to withdrawal from activities, responsibilities, and relationships. This sense of powerlessness resulted in a loss of self-trust and an identity crisis, exacerbating dependence on others and causing various problems for patients and their caregivers. Adverse events, such as betrayal at work, further eroded trust in self and others and led to shame and social avoidance. Caregivers described the patient’s identity change as being mentally and emotionally absent despite physical presence, leaving them feeling unsupported and isolated.

*Patient: “Yes, I became more dependent on others. There are certain things that I used to do automatically, but I can’t do them without extra effort.” (P.5, male, with partner/married, 77 years old)*

*Caregiver: “He was no longer my husband anymore. He was not a father anymore. Such a shame that such a happy person became so negative….” (C.3, female, life partner/spouse, 66 years old)*

Shame and Stigma: Patients and caregivers experienced shame surrounding the patient’s chronic depression, leading to social withdrawal. Patients with sufficient cognitive and emotional capacity and caregivers described playing a role with others to hide negative emotions, avoid judgmental questions about depression, and maintain their social roles. However, this inauthenticity seemed to amplify feelings of shame.

*Patient: “I would do totally nothing … I laid so long in bed. I became an outcast.” (P.26, female, single, 69 years old)*

*Caregiver: “You think that you’re the only one who has it, but every house carries its own cross … But I think our situation is very bad.” (C.1, female, mother, 73 years old)*

Relationship Dissatisfaction: Patients reported issues with sexuality, intimacy, dependency, and feeling misunderstood, leading to overall relationship dissatisfaction. Patients often felt like a burden. Caregivers/partners echoed this dissatisfaction due to the lack of patient support, shared activities, and intimacy. Tension or aggression from the patient toward the caregiver/partner was also noted. Despite contemplating divorce, many partners chose to stay because they valued the long duration of their relationship/marriage.

*Patient: “I find [sexuality] annoying. I feel physically limited and am often tired.” (P.19, female, divorced, with children, 43 years old)*

*Caregiver: “I wanted to leave so many times, but you don’t just throw away a marriage.” (C.3, female, life partner/spouse, 66 years old)*

Family Suffering and Social and Work Problems: Patients and caregivers shared the concept of family suffering due to the patient’s chronic depression. At home, patients described two family scenarios: struggling to manage daily tasks and childcare, causing guilt, or overextending themselves to care for children while neglecting self-care. Caregivers confirmed that the patient’s chronic depression affected their children and the family dynamic. The patient’s lack of self-trust also influenced their adult children’s self-confidence. Moreover, in social and employment domains, patients faced challenges, including difficulty maintaining friendships and work-related issues, like panic attacks at work, lack of understanding from managers, inability to focus on tasks, and fatigue. This highlighted the impact of chronic depression on productivity and social engagement for the patient.

*Patient: “I found it the worse for my children … you’re not there for them, because you can’t function normally at all.” (P3, female, with partner/married, 56 years old)*

*Caregiver: “When I was in puberty, I really wasn’t comfortable with it myself … as a woman. I think you adopt that behavior from your mother, unconsciously.” (C.9, female, daughter, 26 years old)*

Accepting the Role of Caregiver: Caregivers faced challenges in accepting the patient’s chronic depression and their role as a caregiver. In the absence of the patient’s active involvement, caregivers stepped in to manage childcare and household tasks. As the patient’s identity and behavior changed (e.g., became more passive), caregivers also took it upon their shoulders to care for the patient (e.g., speaking for them, offering motivational words). The added strain of responsibilities without the patient’s assistance for a long time took an emotional toll on caregivers, leading to feelings of loneliness, anger, and guilt. Some caregivers, particularly women, became frustrated and blamed the long duration on the patient: they wished the patient would show a desire to improve and fight to get better. This reflected a difficulty to accept the persistence of the depression. Also, accepting their role as caregivers was crucial for reducing distress, shifting their perspective from “feeling forced” to “choosing” to be a caregiver. In our study, caregivers varied in accepting this role.

*Caregiver: “You give up a lot. A whole lot. You get totally nothing for it in return…” (C.3, female, life partner/spouse, 66 years old)*

### Coping with chronic depression

Self-Management Attitudes: Patients and caregivers shared this theme, although their attitudes towards self-management differed. Caregivers consistently held positive, functional attitudes, enabling them to effectively implement self-management strategies. Patients’ attitudes varied, affecting their ability to utilize self-management techniques and their confidence in doing so. Some patients successfully implemented strategies and recognized benefits, while others struggled due to, for instance, fatigue and lack of insight. Some attempted self-management but struggled to apply appropriate techniques or experienced little benefit. Finally, some patients found self-management impossible and chose not to use these coping techniques. Overcoming these challenges requires patients to perceive the benefits, build confidence, and address coping complications.

*Patient: “Self-management is not for me … If my body stops, then my mind does too. I don’t really speak about self-management.” (P.19, female, divorced, with children, 43 years old)*

*Caregiver: “I imagine that [self-management means that] I can take care of myself, that I actually do it, and that I reward myself if I deserve it.” (C.4, male, life partner/spouse, 73 years old)*

Self-Management Strategies: Patients and caregivers shared this theme, with considerable overlap in their strategies. In general, focusing on hope and cultivating positivity resonated with both groups. Common strategies included undertaking positive activities, making mental adjustments (e.g., accepting the depression situation, adjusting expectations, counting blessings), setting boundaries, seeking out social support, and getting professional support. However, patients emphasized simplicity in their choice of activities (e.g., walking, gardening, painting), which was likely to mitigate stress, given their low stress tolerance. Also, these were often solitary, body- and energy focused strategies like spending time outdoors, attuning to their bodies, and prioritizing rest. Caregivers, on the other hand, prioritized activities that brought joy and meaning and recharged their energy (e.g., taking photos, going swimming with grandchildren, or going to work). Moreover, caregivers helped patients recognize their own depressive cues and set boundaries in the number and duration of daily activities. Caregivers play a crucial role in the development of personal insight and the management of depressive symptoms for patients (see quotation below).

*Patient: “I do something small that I enjoy, like crocheting or drawing. Everything feels kind of pointless, but doing something simple helps.” (P.19, female, divorced, with children, 43 years old)*

*Caregiver: “I try to offer support if she’s feeling panic or feeling down. I try to motivate her, in either case, and try to help her to do somethings. I think that my tasks are mainly offering support, giving advice, or comforting…” (C.9, female, daughter, 26 years old)*

### (Unmet) care needs 

Desired Coping Support: Patients and caregivers both expressed a need for ongoing professional support in various areas related to coping with chronic depression. This included emotional support, medication management, and communication skills building. Both groups also desired additional psychoeducation about chronic depression to better understand its course and symptom management. Patients specifically sought support for self-management development, assertiveness training, and medication management (e.g., tapering off medication). For daily structure and motivation, some patients sought help from coaches. Patients also mentioned needing assistance with physical comorbidities, work-related challenges (e.g., manager disagreements, finding work), and support for children with psychological issues (e.g., autism, borderline personality disorder). Similarly, caregivers desired communication training, more involvement in treatment decisions, and support in coping with patient relapses and suicidality. They found support from general practitioners insufficient and sought more time with mental health professionals for help, although this could be in the form of an online helpline.

*Patient: “I think that one of the most important things is that I get some support regarding my daughter [with psychiatric issues]. Finally, that’s happening. But that’s asked a lot from me the last several years and I’ve been alone in all of this.” (P.13, female, with partner/married, 41 years old)*

*Caregiver: “I just wanted to know more, to have someone to offer guidance. But no, absolutely nothing.” (C.1, female, mother, 73 years old)*

Coping Complications: Patients and caregivers shared the same theme. It also overlapped with “Struggling with caregiving,” highlighting additional desired needs to support coping. Both groups faced relationship tension, treatment challenges, and the complex role of being both patient and caregiver, considering that a few patients in our study were also caregivers of elderly parents and/or partners. Patients specifically encountered difficulties coping with chronic depression, including psychological and physical comorbidities, limited social support, and mental/cognitive and personality-related challenges (e.g., lack of insight, inability to sense one’s boundaries, perfectionism, or emotional sensitivity). Caregivers struggled with the patient’s prolonged treatment and suicidality. Arranging psychiatric admissions for suicidal patients was particularly distressing for caregivers.

*Patients: “I started to develop an anxiety disorder, and due to that, I noticed I became more regularly depressed.” (P.25, female, single, 67 years old)*

*Caregivers: “He was in and out of the hospital. The third time, he was home, and he was so sad, and I remember, that he was only so tired, and he wanted to die.” (C.1, female, mother, 73 years old)*

## Discussion

### Main results in relation to available literature

Our findings highlight the substantial burden that persistent depressive disorder (PDD) places not only on patients but also on their informal caregivers. Patients with PDD often experience profound hopelessness and powerlessness, leading to loss of identity and trust in themselves and others. In line with our results, De Smet & Meganck (37) described these identity-related changes in personality, interests, and will to live as the patient’s “depressed self”. Moreover, shame and stigma surrounding PDD exacerbate social withdrawal and reinforce the “depressed self”, a sentiment also documented in previous research in depressed adults in primary care (49, 56).

Similarly, caregivers also experience a high burden of disease, which deserves more attention. Caregivers often report feeling overwhelmed, frustrated, and isolated. In some cases, relational tension escalated into conflict or aggression, a finding which was corroborated by female caregivers of males with (acute) depression in a previous study (79). In addition, many caregivers are seriously troubled by the patient’s suicidal behavior. To better cope with the high levels of (dis-)stress caused by the patient’s chronic depression, caregivers may seek out psychological or pharmacological treatment for their own emotional symptoms. Moreover, due to the patient’s increasing dependency, caregivers (especially spouses/life partners) become the metaphorical hands and mouth of the patient. The interdependence of this relationship shows the profound impact on caregivers and reinforces the need to treat the patient and caregiver as a system (28, 38). Our results suggest, however, that the system is not adequately addressed in specialized mental healthcare.

Regarding self-management, patients vary in their attitudes (positive and negative) towards self-management, suggesting varying levels of insight, knowledge, and self-efficacy. On the other hand, caregivers readily recognize the benefits of self-management strategies and consistently employ them for self-care and self-preservation (79). For patients, negative attitudes towards self-management might be due to the substantial effort demanded from the patient, where it has been reported that physical, emotional, and environmental factors (e.g., fatigue, hopelessness, stigma, respectively) often prevent optimal engagement in self-management practices (80, 81). Patients require intrinsic motivation, insight, self-efficacy (i.e., confidence in their ability), and emotion management to effectively utilize self-management strategies, and this process takes time and effort (53, 82, 83).

Both patients and caregivers employ strategies such as engaging in positive activities, setting boundaries, and seeking social support, with patients focusing on simple, solitary activities (e.g., going for a walk) and caregivers on meaningful and joyful activities to recharge energy. The identified self-management strategies are in line with literature on self-management in adults with depression or chronic somatic disease (19, 43, 45, 46, 56, 84). Our results show support for the notion that simple self-management strategies (e.g. getting out of the house, getting enough sleep, engaging in physical activity), may be relevant for all phases of depression (42, 85).

The reported care needs of PDD patients align with those of individuals with depression in online forums, which included medication advice, professional treatment, understanding depression, disclosure and stigma, and help with comorbid mental health problems (80). Patients primarily seek ongoing professional guidance for emotional support, medication management, PDD psychoeducation, and communication skills building. Caregivers desire communication training and psychoeducation on chronic depression and suicide prevention. Specifically, they request more involvement in understanding treatment decisions and learning coping strategies for patient relapses and suicidality. Psychoeducation has been shown to be a consistent unmet need in managing chronic depression and should be more widely implemented for patients and caregivers (55).

### Practical implications and future research

In our study, the high burden of disease of PDD was coupled with a relatively high need for care, including a desire for continued mental healthcare and care in social domains. Given the increasing mental health expenditures and limited therapist capacity (86–88), it is essential to evaluate the financial sustainability of desired care for patients and caregivers. Currently, patients with PDD utilize more mental healthcare than those with acute depression, and despite long-term care and varying therapeutic approaches, many experience poor response to treatment and do not reach remission or recovery (4, 15, 89, 90), resulting in frustration and hopelessness (91). This disrupts patient empowerment and makes it more difficult to promote behavioral change in further treatment. Additionally, research has clearly demonstrated the recurrent and/or chronic nature of depression (8). Therefore, functional recovery/self-management should be discussed with patients, early in treatment, if signs of symptom recovery are not immediately apparent.

First, we recommend that mental health professionals guide the patient towards an attainable recovery-related goal that provides a daily sense of wellness and purpose (e.g., ability to care for children, relatives, or pets; maintaining a friendship; or retaining a job or volunteer work). Having this positive future perspective allows the patient to act and think in a way that facilitates a sense of personal empowerment (56), and may increase the patient’s intrinsic motivation. This personal recovery-related goal, combined with self-management skills, could also be used as a satisfactory point to evaluate treatment completion.

Second, mental health professionals can facilitate self-management skills acquisition by changing negative self-management attitudes (i.e., believing it is useful and possible). This can be achieved through teaching a wide range of self-management strategies, which empowers patients to create their unique toolkit of activities and strategies (55). Mental health professionals may also help the patient gain insight into personal patterns and choice of self-management strategy by tracking mood, energy levels, stress and events using a (digital) tool, such as Pacifica (a phone app that combines cognitive behavioral therapy and mindfulness) (92). Also, adjusting patient expectations regarding activities (e.g., what is possible and not possible for the patient) and focusing on success moments could help the patient work towards a softer self-concept and greater self-confidence in employing self-management strategies (37, 41, 56) Finally, complementary digital/eHealth programs (e.g., online PPEP4All-PDD) and online peer communities (e.g., Depression Connect) can provide cost-effective support for patients, addressing desired care such as communication skills and social contact (57, 93), while increasing internal motivation and reducing stigma (43). Currently, digital/eHealth programs are lacking for PDD, and more research is needed into developing these programs.

Third, involving the caregiver early in treatment may empower the patient and caregiver as a system and may, in turn, reduce dependence on the mental healthcare system. We recommend that mental health professionals perform an inventory of caregiver’s questions or needs. Mental health professionals may consider providing system therapy or (online) psychoeducation programs that focus on the course of PDD, dealing with suicidality, and effective communication. For caregivers, the caregiver-specific ‘*caregiver suicide education program*’ may be beneficial in helping caregivers cope with patient suicidality and involve them in developing/understanding the patient’s suicide prevention plan (94). This may reduce the burden of PDD for caregivers and improve patient outcomes. More research is needed to develop and test communication-building programs and psychoeducation programs that focus on the course of PDD for caregivers.

### Strengths and limitations

To our knowledge, this is the first qualitative study to provide a multi-perspectives account from both patients and informal caregivers regarding their self-management experience/coping and needs concerning PDD. Our qualitative study had some particular strengths. First, the qualitative interviews allowed participants to provide an in-depth description of their personal experiences with living/coping with PDD. In addition, participants provided rich data regarding coping barriers/complications and care needs. Also, we included both adult and elderly depressed patients from specialized mental healthcare, which provided further insight into the use of and attitudes towards life-span self-management. Finally, our study provided the option for participants to verify their transcripts, which could correct any inaccuracies, while the second author acted (IC) as an auditor for the selection of the final themes.

There are also limitations to this study. First, the sample used in this study included patients who were mainly Dutch and highly educated, which limits the generalizability of the results. Second, we came to include fewer caregivers than patients in the study, despite our intention include a purposive sample with maximum sampling. The sample of caregivers is limited, and we only included 3 caregivers with other types of relationships than a life partner (i.e., daughter, mother, friend, see **Table 1**). While all of the caregivers we interviewed reported largely the same experiences or themes, we may have not fully captured the experiences of caregivers with other types of relationships to the patient with PDD. Third, due to safety concerns during the COVID-19 pandemic, we conducted some interviews exclusively by telephone. However, this procedure was previously enacted while avoiding negative consequences in terms of quality and participant-satisfaction. Fourth, while we aimed to plan all qualitative interviews prior to the start of the self-management intervention, there were 3 participants who could only be reached for the qualitative interview after starting the PPEP4All-PDD program. It is possible that this may have led caregivers to be more aware of what self-management means. However, this was balanced by the other caregivers who had not yet started the self-management program. Also, caregivers’ long-term involvement with the patients may have meant that they sought out knowledge and tools to deal with the patient’s depression. Caregivers may have reported having knowledge of self-management at baseline for this reason.

## Conclusions

This qualitative study emphasizes the profound burden and impact of PDD on both patients and their informal caregivers. Given patients’ ambivalent attitudes toward self-management and the barriers they face in coping effectively with PDD, we recommend that healthcare professionals facilitate patients in the process of development and maintenance of self-management strategies. Ideally, attention for self-management should start early in the treatment process to promote behavioral change and skills acquisition and prevent chronic dependency on care. In addition, informal caregivers should be better professionally supported, in particular in dealing with suicidality of patients, for example, by means of targeted psycho-education or participation in a caregiver-specific self-management program, which may both reduce the burden of PDD for caregivers and improve patient outcomes.

## Data Availability

Datasets generated and/or analyzed during the current study will be pseudonymized and stored on an online Dutch meta-data catalogue called the Data Archiving and networked Services (DANS, www.dans.knaw.nl), according to the funding sponsor policy, with access limited to a designated team within the Department of Psychiatry of the Leiden University Medical Center. External researchers may get access to the final trial dataset from the designated team on reasonable request. The (intellectual) property rights with regard to the generated data will reside at the Leiden University Medical Center (Department of Psychiatry). Anonymized results will be published in peer-reviewed journals and presented in international conferences.
