# Supplementary material for: Self-management strategies and care needs of patients with persistent depressive disorder and their informal caregivers: a multi-perspectives qualitative interview study
Source: Front Psychiatry. 2025 Jun 20;16:1505396. doi: 10.3389/fpsyt.2025.1505396 (PMC12226563; doi:10.3389/fpsyt.2025.1505396)
Supplement: Supplementary file 1 [file DataSheet1.pdf]

SUPPLEMENTARY MATERIAL

Table A. Topic guide for **PATIENTS** in the qualitative interview study

	**Topics and main questions**	**Probes and prompts**
**A**	**Experience of chronic depression**	
A1	Can you tell me what it's like for you living with long-term depression?	*How long have you experienced depression?*<br>*What was the cause of the depression?*
A2	How has your life changed due to depression?	*Which changes were there in your life?*<br>*What made those changes happen?*<br>*General prompts: Daily activities, health-related matters such as medication, diet, alcohol use and/or drugs, body/energy levels, study or work, relationships, religion/spirituality.*
**B**	**Coping with chronic depression**	
B1	What do you do to feel a bit better when you are feeling depressed?	*How do these things help?*
B2	Which family members or friends support you the most?	*How do they help or support you?*<br>*What could they do to help more?*
B3	How do you know if something or someone is helping or not?	*How could you experience or see that it is helping you?*
B4	When you're going through a particularly bad time, how do you handle this?	
B5	When things are going well, what do you do to keep that going?	*What are helping things for you then?*
B6	Some people talk about self-management or being in charge of their own life. What does it mean for you?	*How in charge of your life are you?*<br>*What gives you hope in your life?*
**C**	**(Unmet) Care needs**	
C1	What kind of activities can you do less well or not anymore due to your depression?	
C2	Are there any other problems that you experience besides depression, for which you would like to receive help or support?	*Can you please tell me more?*<br>*General prompts: Physical problems; social problems; problems with daily activities (e.g., self-care, eating, cooking, shopping, cleaning, possible care for children/housemates); other.*

**D**	**General**	
D1	Is there anything else you would like to add that we may not have asked?	*What was that like for you?*<br>*Can you tell me more about that?*<br>*And then what happened?*<br>*Have you mentioned everything you'd like to?*

Note. Partly based on topic guide in Chambers et al., 2015.

Table B. Topic guide for **PARTNERS/CAREGIVERS** in the qualitative interview study

	**Topics and main questions**	**Probes and prompts**
**A**	**Experience of chronic depression**	
A1	Can you tell me what it's like for you to live with someone with long-term depression?	
A2	How has your life changed due to depression of the patient?	*Can you please explain further?*<br>*General prompts: Daily activities, health-related matters such as medication, diet, alcohol use and/or drugs, body/energy levels, study or work, relationships, religion/spirituality.*
**B**	**Helping a loved one with chronic depression**	
B1	How do you try to help a person with depression?	*How do these things help?*
**C**	**Coping with chronic depression**	
C1	Some people talk about self-management or being in charge of their own life. What does it mean for you?	*How in charge of your life are you?*<br>*What gives you hope in your life?*
C2	Caregivers can also practice self-management to cope with stress. How do you use self-management in your daily life?	
**D**	**(Unmet) Care needs**	
D1	What kind of activities can you do less well or not anymore due to caring for a person/the patient with depression?	
D2	Where do you experience stress due to caring for a person with depression?	*Which problems do you experience?*<br>*Can you please tell me more?*<br>*General prompts: Physical problems; social problems; problems with daily activities (e.g., self-care, eating, cooking, shopping, cleaning, possible care for children/housemates); other.*
D3	Where would you like to receive more support?	
**E**	**General**	
E1	Is there anything else you would like to add that we may not have asked?	*What was that like for you?*<br>*Can you tell me more about that?*<br>*And then what happened?*<br>*Have you mentioned everything you'd like to?*

Note. Partly based on topic guide in Chambers et al., 2015.

CODING AND ANALYSIS PROCESS

Coding was performed according to qualitative research guidelines for Grounded Theory (Bruscaglioni, 2016; Strauss & Corbin, 1990; Strauss, 1998). In general, two investigators independently coded the information and combined the codes into meaningful groups. Subsequently, these groups were categorized in this study into themes, to build a model concerning self-management/coping with PD (or patient with PD). Participants' quotes were used to illustrate this developed model (e.g. Kraaijvanger et al., 2017). For the quotes in our study: see Tables C (patients) and D (caregivers) of the Supplementary Material.

More specifically, our coding and analysis process started with *open coding*, which included rereading the transcript a few times to familiarize oneself with the data and summarizing the data. During open coding, we examined the transcripts line by line, and we identified and labeled the parts of the interview related to the research question and sensitizing concepts with (meaning) codes. The data were coded "in vivo", as much as possible, to maintain important relevant expressions used by the participant.

During *axial coding*, we started the process of finding patterns in the codes, looking for similarities, creating (sub)groups of codes, and considering their relations. Codes were rephrased, if necessary, combined, or organized into thematically-connected groups (i.e., subthemes and main themes). In this step, we used the constant comparison analysis method, continuously interpreting and comparing (A) the codes within one interview, (B) codes and (sub)groups between patient partner/caregiver interviews, and (C) codes and (sub)groups between partner/caregiver interviews. Eventually, we also compared (D) codes and (sub)groups between patient and caregiver interviews.

During *selective coding*, we developed a core theme for patients and for caregivers, separately, building the subthemes and main themes around them. The second author audited the core, main, and subthemes by asking critical questions regarding the rationale behind the interpretation process in regards to the research questions and sensitizing concepts. Additionally, this method was used to consider the relational links between patient and caregiver themes and to refine the formulation of themes in general.

Table C. Patient super-ordinate themes and subthemes.

**Core theme**				
**FALLING INTO THE SEA OF CHRONIC DEPRESSION AND STRUGGLING WHILE LEARNING TO SWIM THE ENDLESS WAVES**				
**Sensitizing concepts**	**Main themes**	**Subthemes**	**Example(s) of Codes**	**Example(s) of Interview Content (Quotes)**
**Experience of chronic depression**	[A] No control over my chronic depression	[A1] Depression is sudden and unexpected	Feeling helpless; no control; being overcome; unexpected depression-free periods; feels sudden.	*"Depression just happens; it overcomes you." (P.20, female, single, 69 years old)*<br><br>*"I can't recognize when I start getting depressed again. It's very difficult, but you can't do anything about it." (P.4, female, single, 72 years old)*
		[A2] Depression fluctuates yet persists	Comes in waves; impacted by weather; link time of day and severity; winter was worse; chronic nature.	*"It's now less present, but the danger is always there." (P.3, female, with partner/married, 56 years old)*<br><br>*"If I have to describe my mood, there is always a light undertone of depression." (P.10, female, with partner, 65 years old)*
		[A3] Depression is very heavy	Difficult; heavy; negative feelings (sadness; fear; loneliness; inferiority; helplessness); struggle; difficult to explain	*"[Anything I learn from depression] does not outweigh the weight of the depression. I have to say, depression is really a terrible disease." (P.3, female, with partner/married, 56 years old)*<br><br>*"I told my husband that it feels like there is a black sheet that hangs over everything. I don't remember when I really felt happy. It is just really heavy." (P.16, male, single, 64 years old)*<br><br>*"I never really feel depression-free. It's always hanging around. I'm never really happy about anything. But I'm also never really that sad about anything. You miss a lot of your life this way." (P.10, female, with partner/married, 65 years old)*

		[A4] Depression lowers my stress/emotional capacity	Too many stimuli; quickly overwhelmed; high stress; tension becomes aggression; emotional dysregulation; anger.	*"My girlfriend and I tried to live together, but that didn't work out. It overwhelmed me and made me feel very tense." (P.28, male, single, 40 years old)*<br><br>*"I think I could be pretty ugly. Just like I said, because of all that agitation that I felt. I just reacted badly." (P.3, female, with partner/married, 56 years old)*
	[B] Depression changed my self-concept (more passive)	[B1] Loss of will to live	Suicidal thoughts; belief that life has no hope; neglecting self; doubts about living.	*"There's nothing that gives me hope for the future, unfortunately." (P.27, female, single, 57 years old)*<br><br>*"If it's not going well, then I just want to be alone, and I don't see the value in living anymore. I also don't seek out any help." (P.28, male, single, 40 years old)*
		[B2] Loss of interest and identity	Apathy; no desire/interest for anything; identity replaced by depression; emptiness.	*"Depression takes over your personality." (told by caregiver about patient, C.9, female, daughter, 26 years old)*<br><br>*"I'm totally not like that, but you notice that your whole personality has changed." (P.1, male, single, 48 years old)*<br><br>*"I don't know how it would be without my depression." (P.21, female, with partner/married, 50 years old)*
		[B3] Loss of trust in self	Difficult to trust self; dependency on others; low self-esteem.	*"You just lose all trust in yourself." (P.1, male, single, 48 years old)*<br><br>*"For yourself, you think it's not worth it to do anything." (P.1, male, single, 48 years old)*<br><br>*"Yes, I became more dependent on others. There are certain things that I used to do automatically, but I can't do them without extra effort." (P.5, male, with partner/married, 77 years old)*

		[B4] Loss of trust in others	Can't trust others; disappointment by friends, family, or co-workers; others are cruel; loss of trust after traumatic experience; mistrust/suspicion; hate.	*"You trust in humanity actually. If you've worked for a company for 15 years, then you feel betrayed and disappointed."* (P.1, male, single, 48 years)<br><br>*"You see so many people around you just disappear. You no longer hear from them, or see them, and I think, 'No, sorry.' That's not real friendship."* (P.3, female, with partner/married, 56 years)<br><br>*"I just don't like people. They disappointed me so many times. I have experienced so much misery that I actually don't have any desire to have contact with people."* (P.23, male, divorced, with children, 55 years old old)
	[C] Experiencing shame and stigma (about depression)	[C1] Feeling misunderstood	Misunderstood; lack of understanding from environment; negative expectations; others can't deal with me; imagining what others are thinking without asking; others don't understand my depression	*"It's difficult to see other people, considering what they think about the situation. You go on imagining what they're thinking, even though you really don't know if it's true."* (P.1, male, single, 48 years old)<br><br>*"[Depression] is too complex for others to understand. I couldn't really explain it. I thought I was an oddball."* (P.26, female, single, 69 years old)
		[C2] Withdrawal and avoiding the topic (depression)	Isolating self; avoidance; becoming an outcast	*"I didn't do anything, like going out or meeting up with people. Didn't cook. Didn't pick up the phone. I was in my bubble."* (P.8, female, single, 72 years old)<br><br>*"I would do totally nothing, then I just got so dirty, and I then couldn't even walk, because I laid so long in bed. I became an outcast."* (P.26, female, single, 69 years old)
		[C3] Hiding my self	Shame; playing a role; you can't really show yourself; I can't or don't want to talk about my depression with others	*""I always kept it hidden, like, colleagues, or neighbors, or even family."* (P.26, female, single, 69 years old)<br><br>*"You develop a kind of role play, and that is exhausting, because then you're not yourself."* (P.26, female, single, 69 years old)

	[D] Depression strained my romantic relationship	[D1] Sexuality/intimacy issues	Sexuality; communication issues; less sexual satisfaction	*"And he'd always say something like, 'Ah come on!', even though it just didn't work, in that area. We were less intimate." (P.13, female, with partner/married, 41 years old)*<br><br>*"I find [sexuality] annoying. I feel physically limited and am often tired." (P.19, female, divorced, with children, 43 years old)*
		[D2] Change of role and dependency	Change in relationship role; relationship dependency; feeling weaker	*"It had an impact on [my husband]. It is different than if you are standing on equal ground." (P.6, female, with partner/married, 67 years old)*<br><br>*"I became so much more dependent on others. There are certain things that I used to do routinely, and now I really have to push myself to do things." (P.5, male, with partner/married, 77 years old)*
		[D3] Relationship dissatisfaction	Relationship tension; partner also has a diagnosis; partner is stressed/frustrated; partner needs to carry more of the load; partner is old; partner can't help me; feeling like you disappoint others	*"I think that I felt very misunderstood, and that led to tension [in the relationship]." (P.13, female, with partner/married, 41 years old)*<br><br>*"I felt guilty. I don't think I ever did anything wrong. But I let others down, that's clear. And that really bothered me. Actually, after 10 years old of marriage, out of respect for my husband, I decided to divorce him." (P.26, female, single, 69 years old)*
	[E] Depression strained other relationships	[E1] Loss of social contacts	Loneliness; can't ask friends for help; limited in making friends; pushing others away; if things aren't going well, I'm not aware; I limit contact with others	*"Sometimes people ask to come over. But I think it's hard to ask for help." (P.28, male, single, 40 years old)*<br><br>*"[Depression] limits you in your social life. As soon as people get closer, you want to push them away. It's really hard to make real friendships." (P.26, female, single, 69 years old)*
		[E2] Change in caring for children	Struggling with children; can't ask children for help; children lead to	*"Well, you know, I found it the worse for my children. Actually, you're not there for them, because you can't*

			extra stress; need help from partner with children; depression impacts caregiving	*function normally at all." (P3, female, with partner/married, 56 years old)*<br><br>*"I don't know how long it will take before the pain of my depression becomes greater than the love for my children and my grandchildren." (P.23, male, divorced, with children, 55 years old old)*
		[E3] Loss of work and colleagues	Loss of work; no work, due to depression, physical issues or retirement.	*"There was a time that I tried to work, but I needed to let it all go. I just couldn't do it." (P.6, female, with partner/married, 67 years old)*<br><br>*"I was able to carry on working for 3 years. But yeah, your condition becomes not really great [after depression] so you can't even work a full day." (P.2, male, divorced, 66 years old)*
**Coping with chronic depression**	[F] Self-management attitudes	[F1] Believing self-management possible and/or useful (+)	Self-management gives stability; gives fulfilment; helps to learn from depression; I can control my depression; recovering my life; I can talk when I start to feel better; I can seek help when I start to feel better; it's a process but it's possible; I can structure my life; it's necessary; I am the boss of my life;	*"I can plan my life the way I want. It does not have to be a lot, as long as I have a nice life." (P.3, female, with partner/married, 56 years old)*<br><br>*"You have the ability to decide how you live." (P.20, female, single, 69 years old)*<br><br>*"I am indeed the boss of my own life. I know it well." (P.4, female, single, 72 years old)*<br><br>*"But that self-management, it falls through your fingers when you're depressed. And you need to pick it up, and you need to really fight for it." (P.6, female, with partner/married, 67 years old)*
		[F2] Believing self-management impossible and/or useless (-)	I have no positive activities; I have no control over my thoughts; it's not realistic to be the boss; it's so very hard to live with depression; it is	*"Self-management is not for me…If my body stops, then my mind does too. I don't really speak about self-management." (P.19, female, divorced, with children, 43 years old)*<br><br>*"It's not realistic to think you can be the director of your life." (P.25, female, single, 67 years old)*

			too hard to cope (for yourself); you can only go with the flow	
		[F3] Willing and ready to use self-management (+)	Others can't suffer on my behalf; you have the power over your life; I can act but it doesn't mean I'm doing well	*"I do the things I need to do and that just goes on. It's just that I don't really experience any pleasure or satisfaction from it." (P.27, female, single, 57 years old)*<br><br>*"You need to realize that things aren't going well and then take action."* (P.21, female, with partner/married, 50 years old)
		[F4] Unwilling or unable to use self-management (-)	I can't act on self-management; can't handle things; feeling blocked; struggling so much to act; no energy for self-management; procrastination	*"I have ideas for activities, but I can't bring myself to do them."* (P.46, female, single, 69 years old)<br><br>*"I can't ask for help. I can't get started."* (P.28, male, single, 40 years old)<br><br>*"In bad moments, I am totally not aware. I'm prepared to isolate myself from everything and everyone."* (P.23, male, divorced, with children, 55 years old old)
	[G] Self-management strategies	[G1] Doing practical activities and going outside	Practical matters; work, cleaning your home; going outside; pushing yourself to go out; doing groceries; giving life rhythm and structure; focus on the basics; plan your day.	*"And what I do is obligate myself to go outside at least 1 time a day, to go buy groceries, for instance."* (P.11, male, with partner/married, 60 years old)<br><br>*"I have a walking buddy, and I go on a walk 2x per week with that person, for a few hours, and that helps."* (P.5, male, with partner/married, 77 years old)<br><br>*"Set routines. Plan a few things for each day, so you don't wake up and think, 'well what am I going to do today?'"* (P.1, male, single, 48 years old)
		[G2] Doing simple positive activities	Hobbies; positive activities (gardening, crocheting, watching TV, reading, biking, meditating, puzzles,	*"Gardening gives a sense of fulfilment. There's nothing there to tell you how you need to do it. And I think that's great."* (P.1, male, single, 48 years old)

			music, painting); laughing/humor; walking.	*"I do something small that I enjoy, like crocheting or drawing. Everything feels kind of pointless, but doing something simple helps."* (P.19, female, divorced, with children, 43 years old)
		[G3] Making mental adjustments	Acceptance of situation, diagnosis; relativizing; increasing positivity; adjusting expectations; replacing negative thoughts with positive ones; enjoying the little things; recognizing hope; not giving up.	*"You become more realistic. I need to keep an eye on my [thoughts] and not say that I am the only one with big problems. That helps, of course."* (P.2, male, divorced, 66 years old)<br><br>*"It was very difficult, but I went to the [grocery store] a few times, and I feel more confident in it. So now I think, "If I succeeded in this, then I can do other things."* (P.5, male, with partner/married, 77 years old)
		[G4] Setting boundaries and protecting my energy	Avoiding triggers (negative situations or people); distraction from negativity; releasing negative emotions; setting boundaries; asking for space.	*"You know what your sensitivities and triggers are. You need to try to avoid those actually. It doesn't always work. But you know what is possible for you."* (P.2, male, divorced, 66 years old)<br><br>*"I became more direct and I set my boundaries. Till here and no further. And it helps me."* (P.3, female, with partner/married, 56 years old)
		[G5] Focusing on the body and getting more rest	Focusing on eating healthy; taking warm showers; taking your medication; going with the flow; body over mind; exercising; body awareness; getting rest; doing less; taking breaks; recharging; relaxing; mindfulness and breathing exercises.	*"I listen more to my body, by lying down to rest or sleeping. Just being. I give myself 1 to 2 days to process the exhaustion."* (P.19, female, divorced, with children, 43 years old)<br><br>*"Rest is important, just relax."* (P.7, with partner/married, 48 years old)<br><br>*"I wake up. I shower. And I eat. Prepare my food."* (P.4, female, single, 72 years old)
		[G6] Seeking social contact and family support	Asking help from family/friends/partner; getting help from	*"My parents help. They can see in which phase I am. They help, with calling, asking me to do things."* (P.1, male, single, 48 years old)

			maintaining social contact; recognition/understanding from others; getting invitations; getting help with practical things from friends/family; keeping appointments; social life is important	*"I go out or I go see someone. To drink a cup of coffee. Or I call someone every now and then." (P.9, male, with partner/married, 60 years old)*<br><br>*"And yeah, I still try to do at least half a day of volunteer work, somewhere close by." (P.6, female, with partner/married, 67 years old)*
		[G7] Seeking professional support	Help from mental health care; medication (antidepressants) help; mindfulness helps; psychiatric admission was necessary and helpful; various specific treatments; help from other healthcare providers (for finances/debt, showering/self-care, household cleaning; physical therapy); keeping professional appointments	*"I get help from the home care team. They come to help me dress and shower." (P.4, female, single, 72 years old)*<br><br>*"I think that when I did not have medication or any help, that it would have gone the wrong way. Receiving medication was a turning point for me." (P3, female, with partner/married, 56 years old)*<br><br>*"[With the therapist], you have good talks, and you can learn a lot, and she gives you direction and tips, and that helps." (P.5, male, with partner/married, 77 years old)*<br><br>*"I think taking my medication especially helps. That is my sign post. That is started to work. It took time. But now I can concentrate better and I can reflect/think more clearly." (P.1, male, single, 48 years old)*
**(Unmet) care needs**	[H] Desired coping support	[H1] Finding work	Work is desirable; finding work; need help with work.	*"He [the patient] is looking for job, volunteer work, or his friend will look for him." (Mother said about patient, P.1, male, single, 48 years old)*<br><br>*"I would like help with finding other work." (P.21, female, with partner/married, 50 years old)*<br><br>*"I think I really miss having a job. I wouldn't want full-time but working part-time or a number of hours seems all right. I*

				look regularly at job openings." (P.27, female, single, 57 years old)
		[H2] Support with children with psychological or physical issues	No support (children); son has problems; children with psychiatric issues	*"I think that one of the most important things is that I get some support regarding my daughter [with psychiatric issues]. Finally, that's happening. But that's asked a lot from me the last several years and I've been alone in all of this." (P.13, female, with partner/married, 41 years old)*<br><br>*"My daughter also needs an instruction manual. She has borderline and that isn't easy." (P.23, male, divorced, with children, 55 years old)*
		[H3] Help improving assertiveness and communication	Learning how to set boundaries; becoming more assertive; feeling more like myself.	*"I am more the kind of person who listens. But I need to stand up for myself and also talk about myself. To give my own opinion." (P.6, female, with partner/married, 67 years old)*<br><br>*"I can really go over my boundaries. I really struggle with that." (P.12, female, single, 55 years old)*
		[H4] Help with understanding chronic depression	Psychoeducation depression desired; learning more about chronic depression desired.	*"It's a puzzle why I had so many problems with depression in my youth. I want to understand why I got depressed so young." (P.28, male, single, 40 years old)*<br><br>*"It is strange to experience how long it takes you to recognize that there is actually a problem and that you see and feel things differently." (P.8, female, single, 72 years old)*
		[H5] Continued professional support	Missing professional guidance; desired medication controls; desired follow-ups mental healthcare.	*"With help from oxazepam (medication), I can function better societally and socially, but not wholeheartedly…I miss the support from [mental health clinic]." (P.11, male, with partner/married, 60 years old)*<br><br>*"[My therapist] was a psychiatric nurse, and she felt a bit like a friend. She was an open person." (P.3, female, with partner/married, 56 years old)*
	[I] Coping complications	[I1] Pharmacological treatment problems	Hard to find right medication; medication no longer works; side	*"With medication, I noticed that it worked for a while, but then we needed to change to another one. And sometimes had issues with that one, so then you also got depression*

			effects and/or increased severity of depression related to medication; fear of stopping or lowering dosage of medication.	*symptoms, because of the medication." (P.6, female, with partner/married, 67 years old)*<br><br>*"Medication helped, but then after a while, the effectiveness, it just stopped." (P.5, male, with partner/married, 77 years old)*<br><br>*"I'm now stable, luckily, but I heard that I need to take [the medication] the rest of my life, because they are scared that if they lower the dosage or if I stop, that I will relapse." (P.7, female, with partner/married, 48 years old)*
		[I2] Psychological treatment frustrations or doubts	No click with therapist; feeling stuck in treatment; many previous therapies/ therapists; talking to GP doesn't help; programs cut due to cutting costs, therapist stopped treatment.	*"I was referred to a psychiatric nurse specialist. But it's not really helping. I have no click with her." (P.4, female, single and retired, 72 years old)*<br><br>*"I used to receive sport therapy, "Fit Your Body", at the mental health clinic, with people with a psychiatric background. It really helped me but the program was cut out for budget issues." (P.1, male, single, 48 years old)*
		[I3] No social support	No friendships or relationships; no family in the area; no interaction with others; no support; need to depend on yourself	*"I don't have any family or friends who are willing to help me. I've never had that, and I feel very sad about that. It hurts a lot." (P.20, female, single, 69 years old)*<br><br>*"I've always been by myself. I've never really had real friendships." (P.2, male, divorced, 66 years old)*
		[I4] Aggression and abuse	Aggression from partner; anger; fights; abuse in relation to others.	*"[My partner] was not consistent in his behavior. Sometimes he got very angry. And because of this, I reached the peak of my depression. It was very bad then." (P.19, female, divorced, with children, 43 years old)*<br><br>*"It was a bad marriage, with emotional and physical abuse. I'm now divorced after seven years." (P.20, female, single, 69 years old)*

		[I5] Psychiatric comorbidity	Anxiety disorder, panic; anxiety/panic attacks; hypomania; ADHD; (past) psychosis; alcohol and drug use.	*"I started to develop an anxiety disorder, and due to that, I noticed I became more regularly depressed."* (P.25, female, single, 67 years old)<br><br>*"Before, when I was really down, I was smoking weed all day. It helped."* (P.12, female, single, 55 years old)
		[I6] Physical comorbidity	Physical issues limit me; weight issues; physical exhaustion; physical pain; stomach problems; high blood pressure; older age; epilepsy; migraines/headaches.	*"I had years of terrible chronic pain, in my back."* (P.14, female, single, 65 years old)<br><br>*"They said I had very high blood pressure, so I immediately received a blood pressure reducer. I noticed that I really didn't feel well."* (P.25, female, single, 67 years old)
		[I7] Caregiving challenges	Caring for family members; children have health issues; children have psychiatric issues; grandchildren ask a lot of energy.	*"Life is giving and caregiving; taking care of everyone in your life. My mother was sick. Then I met my man, and I needed to care for him. Then I had two children. And finally, I went back to work in healthcare."* (P.4, female, single, 72 years old)<br><br>*"Yes, it is complicated and difficult. He (husband) has been sick for a long time."* (P.4, female, single, 72 years old)<br><br>*"I don't think that others should suffer because of my depression. So, I continue to care for others, cook food, and do the things for others. But if I, for example, make lunch for them, then I eat with them. But if they already take their lunch boxes with them, then I don't make a lunch for myself."* (P.21, female, with partner/married, 50 years old)
		[I8] COVID-related challenges	Affected availability of (volunteer)work; affected rules at (volunteer)work; greater feelings of loneliness; fear of getting sick; inability to arrange face-to-face appointments	*"During the corona situation, the idea that you couldn't do anything gave me a miserable feeling. I felt lonelier because of it."* (P.27, female, single, 57 years old)<br><br>*"I did a lot of volunteer work, but because of the corona crisis, that came to a stop. So now I don't have any*

			with therapist; mental health programs prematurely stopped.	*distraction. How am I supposed to get through a day?"* (P.20, female, single, 69 years old)
		[I9] Difficulty processing loss	Death of loved ones; loss of significant relationships; loss is too painful	*"After 2 years after the death of my mother, I became depressed."* (P.6, female, with partner/married, 67 years old)<br><br>*"Well, my life was changed after my partner died. I was so afraid for this, because then you don't have your best friend anymore, you know? Then you need to do everything alone."* (P.4, female, single, 72 years old)
		[I10] Personality-related vulnerabilities	High standards (strict on self); perfectionism; introversion; sensitivity; people-pleasing	*"I'm hard on myself."* (P.20, female, single, 69 years old)<br><br>*"I was a very sensitive girl, and my mom needed to vent sometimes, and she always told me [her problems]. That was probably not good."* (P.14, female, single, 65 years old)
		[I11] Mental blocks to getting started	Difficulty accepting situation or diagnosis; lack of insight; don't know when things are getting bad; can't get started; doing nothing; don't know what I want; don't feel my boundaries	*"I can't sense my boundaries."* (P.12, female, single, 55 years old)<br><br>*"It takes long to recognize that there is an actual problem and see and feel things differently."* (P.28, male, single, 40 years old)

Table D. Partner/caregiver super-ordinate themes.

**Core theme**				
**JUMPING INTO THE SEA OF CHRONIC DEPRESSION TO HELP THE PATIENT AND NEEDING TO REGULARLY RETURN TO SHORE FOR AIR**				
**Sensitizing concepts**	**Main themes**	**Subthemes**	**Example(s) of Codes**	**Example(s) of Interview Content (Quotes of Caregivers)**
**Experience of chronic depression for partner/caregiver**	[A] Chronic depression changed the patient	[A1] Missing the patient	Missing the (old version of) partner; not the same person anymore; partner was a fun person	*"Before he never did that. [My husband] always took the lead, and he'd say, 'No, you don't have to worry about that.' But that's gone. And I realize that more and more as time passes." (C.4, male, life partner/spouse, 73 years old)*<br><br>*"He was no longer my husband anymore. He was not a father anymore. Such a shame that such a happy person became so negative, someone who used to be so busy." (C.3, female, life partner/spouse, 66 years old)*<br><br>*"He went deeper and deeper into a hole. In his whole daily life, and with his behavior toward us, toward me and his son." (C.3, female, life partner/spouse, 66 years old)*
		[A2] Feeling unsupported by the patient	Feeling ignored; not supported; feeling alone; separate lives; can't talk to patient	*"[The patient] has so much sympathy for other patients. He even told [another patient], 'You may call me whenever.' Just a kind of father-like support. As a wife, you sympathize, but then, I think, 'What about me?'" (C.3, female, life partner/spouse, 66 years old)*
	[B] Feeling powerless to patient's depression	[B1] Depression is sudden and unexpected	Depression is unpredictable, unexpected, irrational	*"It is just really hard. In the beginning period you are just trying to figure out what's going on. You deal with it every day." (C.3, female, life partner/spouse, 66 years old)*<br><br>*"It's difficult, and what makes it difficult is that it is really unpredictable. You don't understand it; I didn't*

				understand it. And that led to a lot of conflict. I thought, 'what is this, what is happening?'" (C.8, female, life partner/spouse, 74 years old)
		[B2] Depression is hard to recognize or accept (the chronic depression)	Can't recognize signals; can't accept the changes	*"At the beginning you don't know a lot about it and you just try and see what helps the person."* (C.6, female, good friend, 62 years old)<br><br>*"We didn't know what we were going to deal with. Yeah, all three of us did not know."* (C.3, female, life partner/spouse, 66 years old)<br><br>*"It's hard to understand why the depressed person doesn't do more to get passed the feeling, because that person knows that staying in bed all day won't help, and yet she does it."* (C.6, female, good friend, 62 years old)
		[B3] Feeling desperation and worry	Desperation; worrying so much; sleep problems	*"I need to deal with this 24-hours a day!"* (C.3, female, life partner/spouse, 66 years old)<br><br>*"In those times, you are so desperate. You don't know what you need to do."* (C.1, female, mother, 73 years old)
		[B4] Experiencing low mood and sadness	Feeling sad; feeling depressed; depression affects my mood; I have depression	*"It dominated my life. I was crying so much outside."* (C.3, female, life partner/spouse, 66 years old)<br><br>*"It is, of course, really heavy sometimes. It also affects my own mood."* (C.9, female, daughter, 26 years old)
		[B5] Needing professional help and/or antidepressants	Seeking out own help; sleep medication didn't help	*"And earlier, in the time of those hospitalizations, I couldn't function; I was shaking all day."* (C.1, female, mother, 73 years old)<br><br>*"I struggled with his depression, and as a mother, then you can't function. I took medication before, for that, Venlafaxine."* (C.1, female, mother, 73 years old)

				"She was suddenly completely crazy. She threw a package at me. And I said, I can't take this anymore. I couldn't calm her down. So I called 112." (C.2, male, life partner/spouse, 64 years old)<br><br>*"I took pills, but they didn't work. So I stopped. So I just need to suffer through life, because I didn't have any benefit from it." (C.2, male, life partner/spouse, 64 years old)*<br><br>*"I've had a few times where I think, 'Man, what now?' I had a really terrible period, that I felt totally lost. And I went to the doctor, because I felt done with life. And I feel really sad about that." (C.5, male, life partner/spouse, 54 years old)*
	[C] Caregiving is sacrificing without thanks	[C1] Adjusting your life for the patient	I need to be very careful with patient; don't leave the patient alone too long; being more tolerant and empathetic; need to care for self and patient; sacrificing; caring for patient is thankless work	*"You give up a lot. A whole lot. You get totally nothing for it in return, and they don't realize that. They even put up a Berlin wall right in front of you." (C.3, female, life partner/spouse, 66 years old)*<br><br>*"I changed my schedule so that patient isn't alone too long." (C.2, male, life partner/spouse, 64 years old)*<br><br>*"I could get upset, angry. Then I try to go out the door to try to calm down." (C.5, male, life partner/spouse, 54 years old)*
		[C2] Taking the lead	Feeling responsibility; feeling guilty; speaking for the patient; feeling of justice; patient asking for help; patient needs help; taking the lead is stressful; unequal dynamics	*"Yeah, [the patient] doesn't do it for herself, so I catch myself speaking for her. You don't notice it at the beginning." (C.4, male, life partner/spouse, 73 years old)*<br><br>*"It is frustrating, especially if you have been productive all day and then you come home, and you see that someone did very little, even though you yourself are*

				tired from work, And, for example, the dishes haven't been done. Yeah, those are frustrating moments." (C.9, female, daughter, 26 years old)<br><br>*"The dynamics have changed. Earlier it was always equal, but I need to be careful that I do not become a dominant figure in her life. I am, in fact, but maybe not in attitude, but in daily life, I make 80% of the decisions." (C.4, male, life partner/spouse, 73 years old)*
		[C3] Exhausted by effort and lack of help	Helping patient is exhausting; patient does nothing/too little; exhausted; wanted to leave so many times	*"If you can't vent to someone, then you won't last long. Those women in a situation with a depressed husband, who have anyone to vent to, family, friends, or whoever, they can't deal with it all. I can understand that. They just can't handle it." (C.3, female, life partner/spouse, 66 years old)*<br><br>*"I wanted to leave so many times, but you don't just throw away a marriage." (C.3, female, life partner/spouse, 66 years old)*<br><br>*"I can't take it - If [he doesn't] want to do anything. If [he doesn't] want to change. Because with depression, you finally get help, you get medicine. But you need to do it yourself. And you need to do your best. You can't just say, I'm going to take this little pill and I'm going to go there to talk about my story. And I will become the same old person. No, you need to do it yourself. That's how it is, right?" (C.3, female, life partner/spouse, 66 years old)*
	[D] Feeling shame about the patient	[D1] Perceived judgment	Shame; perceived judgment; fear what others will think; stigma	*"I'm ashamed what neighbors think of us." (C.2, male, life partner/spouse, 64 years old)*<br><br>*"You think that you're the only one who has it, but every house carries its own cross. Everywhere there is*

				something. But I think our situation is very bad." (C.1, female, mother, 73 years old)
		[D2] Avoiding the topic (of chronic depression) and others	Don't want to talk about it; limiting social contact; don't want to feel crazy; don't want to burden others	*"Two girlfriends knew about it. They just called me. But besides that, you don't really have anyone. Not at all." (C1, female, mother, 73 years old)*<br><br>*"I can't really talk to my family, because it doesn't run in the family. In our family, it just isn't known." (C.3, female, life partner/spouse, 66 years old)*
	[E] Depression hurts the family	[E1] The negativity brings us all down	Difference before and after depression; it influences me and the entire family; whole family gets sad	*"It is damn difficult for all of us. The whole family. They get so sad, so negative! They take down the whole family with them." (C.3, female, life partner/spouse, 66 years old)*<br><br>*"It is really hard sometimes, and it sometimes affects my own mood." (C.6, female, good friend, 62 years old)*<br><br>*"It costs a lot of energy, and it can affect your own mood, so that you also feel less joyful and even develop mild depressive complaints. It's just demotivating to have someone around you all that time that does so little." (C.9, female, daughter, 26 years old)*
		[E2] It left its mark on the children	As son/daughter, I get affected; impact on children; worry about genetics	*"In the beginning, she (patient) worried about everything. I told her, 'Don't worry so much, relax.' It's also better for the kids. Because they see that, too." (C.9, female, daughter, 26 years old)*
**Coping with chronic depression**	[F] Self-management attitudes	[F1] Self-management means directing my own life (+)	There is no 'should' in self-care; I decide my life; I know best what I need	*"I know best how I should handle something and what works best for me." (C.9, female, daughter, 26 years old)*<br><br>*"Nobody tells me what I need to do. I decide 'how' and 'when.' And no one is going to do that for me." (C.5, male, life partner/spouse, 54 years old)*

		[F2] Self-management means taking care of myself first (+)	Caring for self; needing distance as self-care; sustainable energy	*"I imagine that [self-management means that] I can take care of myself, that I actually do it, and that I reward myself if I deserve it."* (C.4, male, life partner/spouse, 73 years old)<br><br>*"I think it is important* to take good care of yourself and live your day-to-day life in a successful way." (C.6, female, good friend, 62 years old)
	[G] Self-management strategies	[G1] Doing positive activities	Doing fun, positive activities like walking outside, swimming with the grandchildren, or photography; enjoying simple activities with the patient; going to the church; exercising; going to work.	*"I do fun things to forget it all. If I'm feeling heavy, then I go out and I do something fun. I exercise a lot. Then it's all tolerable, I think."* (C1, female, mother, 73 years old)<br><br>*"I enjoy taking photos, just* going somewhere to travel, *going for a walk. Those kinds of things."* (C.5, male, life partner/spouse, 54 years old)
		[G2] Making mental adjustments	Not giving up; focusing on hope; maintaining humor in the relationship; counting your blessings; seeing the positive side.	*"I am very down*-to-*earth, or maybe more pragmatic. I just take it day-by-day and if a situation gets more difficult, than I just keep going."* (C.8, female, life partner/spouse, 74 years old)<br><br>*"I can't really complain. I saw a class* photo from high *school and I thought, 'Oh, that person is dead, and that person is dead.' And I am still frolicking around. So yeah, I count my blessings."* (C.4, male, life partner/spouse, 73 years old)
		[G3] Setting boundaries and protecting my energy	Getting air from the situation; protecting self by taking distance; giving your life structure; being kind to yourself	*"I* learned that I need to sometimes take a step back to *protect myself, that I can't always get pulled into the emotional world of someone else, maintaining a healthy distance sometimes."* (C.6, female, good friend, 62 years old)<br><br>*"You need to make sure that you can deal with* [caregiving]. So that means going to bed on time, that

				you eat healthy, but also, that you're kind to yourself. It's okay to buy a bar of chocolate so now and then." (C.9, female, daughter, 26 years old)
		[G4] Learning more about chronic depression	Going online for information; trying to understand situation; looking in book for information; my job gave me prior knowledge about depression	*"I will go online, and I will go searching, 'What did I miss?'…'Would that have affected [the depression]?'" (C.5, male, life partner/spouse, 54 years old)*
		[G5] Seeking social support	Feeling heard and understood; venting; talking is a remedy; friends to relieve your heart	*"I think that the best remedy is to talk about it, to help [the patient] to understand what it does to other person." (C.1, female, mother, 73 years old)*
		[G6] Seeking mental healthcare / professional support	Therapist-client bond; professional guidance helps me not feel alone; treatment helps me to relativize; I need professional help	*"I had a good connection with [the therapist]. You could see that it has a positive effect. If there is a good click with the therapist, then therapy will work better, and more people are willing to go to their therapy sessions." (C.5, male, life partner/spouse, 54 years old)*
**(Unmet) Care needs**	[H] Desired coping support (with the situation)	[H1] (Integrated) professional support	In relation to patient suicide risk; emotional support; continued mental health sessions; improvement of communication	*"I just wanted to know more, to have someone to offer guidance. But no, absolutely nothing." (C.1, female, mother, 73 years old)*<br><br>*"If [the situation] goes too far, I would like to have that life line, like a therapist or someone who helps and can give a tip, like, 'Well, try this out' or 'Try to think about it like this.' That would be great!" (C.5, male, life partner/spouse, 54 years old)*
		[H2] Psychoeducation about patient suicidality	I need to know more for my sanity; mental health doesn't give enough attention; GP doesn't	*"Now and then there are things that come up, and I think, 'I'm going to go look online. How does that work again?' And then you learn something." (C.5, male, life partner/spouse, 54 years old)*

			really listen; what is normal for depression	
	[I] Complications with my coping	[I1] Psychological or pharmacological treatment doubts	(Medication) didn't work for me; treatment wasn't what I expected; believed less in my treatment	*"I lost all faith in [treatment] at the end. I said, I want to cancel because otherwise you just keep going. You don't get better from it.'" (C.2, male, life partner/spouse, 64 years old)*
		[I2] (Passive) aggression by patient	(Passive) aggression from patient; disappointed by patient; relationship tension.	*"Yeah, (the patient) can get physically mean. And you never want that again." (C.3, female, life partner/spouse, 66 years old)*<br><br>*"She got really aggressive at home. And sometimes I couldn't handle it." (C.2, male, life partner/spouse, 64 years old)*
**Helping the patient**	[J] Caring for the patient	[J1] Offering advice, comfort, and motivation	Comforting patient; motivating patient; consider what is best for patient; I keep learning about depression; believing the patient is a good parent	*"If I recognize it, I try to be there for her (patient) and comfort her. If she's depressed and very sad, then I try to listen to her." (C.5, male, life partner/spouse, 54 years old)*<br><br>*"I try to offer support if she's feeling panic or feeling down. I try to motivate her, in either case, and try to help her to do somethings. I think that my tasks are mainly offering support, giving advice, or comforting…" (C.9, female, daughter, 26 years old)*<br><br>*"[The patient] told my husband, 'there's no one who loves me.' So, I sent him a really nice card from the book store, and I wrote, 'Your mother really loves you.'." (C.1, female, mother, 73 years old)*
		[J2] Being his/her voice	Speaking for the patient; standing up for the patient.	*"I try to be helpful and be what the (patient) wants. She can't do it herself, so then I need to speak for her. I went everywhere. Just name it. Every appointment." (C.2, male, life partner/spouse, 64 years old)*

				"I try to understand [the situation]. And if things don't go well with someone, then I personally go and talk to that person [for the patient] and say that it's not okay." (C.2, male, life partner/spouse, 64 years old)
		[J3] Offering invites to activities	Inviting patient; including patient in activities; helping patient relax	*"We invite [the patient] to do all kinds of things. We take him to the theatre. He comes with us on vacation, to France, with our grandchild." (C.1, female, mother, 73 years old)*<br><br>*"We just go everywhere together. You don't have to but you may. That's very important. And that gives her (the patient) some peace." (C.7, life partner/spouse, 53 years)*
		[J4] Helping to recognize depressive cues	Help patient with boundaries; depression prevention by signalling	*"I can signal if there is something wrong with her (patient). I try, of course, to prevent that she doesn't go so deep in that hole, similar to before." (C.5, male, life partner/spouse, 54 years old)*
	[K] Struggling with patient care	[K1] Frustration about patient treatment	Patient treatment takes too long; patient treatment advice doesn't make sense; dissatisfaction with patient treatment.	*"Slowly but surely, he arranged therapy, and that gave me relief. But that didn't really lead to the desired outcome actually, because [the depression] appeared to be a lot heavier than we thought." (C.8, female, life partner/spouse, 74 years old)*<br><br>*"How long are we busy with this? 10 years? 13 years? I don't know anymore." (C.3, female, life partner/spouse, 66 years old)*
		[K2] Fear of suicide/relapse of patient	Fear of suicide; stressed by psychiatric admission of patient; worry about death of patient; worry about relapse.	*"He was in and out of the hospital. The third time, he was home, and he was so sad, and I remember, that he was only so tired, and he wanted to die." (C.1, female, mother, 73 years old)*<br><br>*"And earlier, in the time of those hospitalizations, I couldn't function; I was shaking." (C.1, female, mother, 73 years old)*

				"Those are times I don't want to remember. I can't say, 'It was okay', because it was really not okay. But now we're in another phase, and I hope we never have to experience that again." (C.8, female, life partner/spouse, 74 years old)
